# Exploring substrate–microbe interactions: a metabiotic approach toward developing targeted synbiotic compositions

**DOI:** 10.1080/19490976.2024.2305716

**Published:** 2024-02-01

**Authors:** Bodo Speckmann, Ellen Ehring, Jiaying Hu, Ana Rodriguez Mateos

**Affiliations:** a Evonik Operations GmbH, Creavis, Essen, Germany; b Department of Nutritional Sciences, School of Life Course and Population Sciences, Faculty of Life Sciences and Medicine, King’s College London, London, UK

**Keywords:** Pharmabiotics, synbiotics, metabiotics, next generation probiotics, drug development, prodrug

## Abstract

Gut microbiota is an important modulator of human health and contributes to high inter-individual variation in response to food and pharmaceutical ingredients. The clinical outcomes of interventions with prebiotics, probiotics, and synbiotics have been mixed and often unpredictable, arguing for novel approaches for developing microbiome-targeted therapeutics. Here, we review how the gut microbiota determines the fate of and individual responses to dietary and xenobiotic compounds via its immense metabolic potential. We highlight that microbial metabolites play a crucial role as targetable mediators in the microbiota-host health relationship. With this in mind, we expand the concept of synbiotics beyond prebiotics’ role in facilitating growth and engraftment of probiotics, by focusing on microbial metabolism as a vital mode of action thereof. Consequently, we discuss synbiotic compositions that enable the guided metabolism of dietary or co-formulated ingredients by specific microbes leading to target molecules with beneficial functions. A workflow to develop novel synbiotics is presented, including the selection of promising target metabolites (e.g. equol, urolithin A, spermidine, indole-3 derivatives), identification of suitable substrates and producer strains applying bioinformatic tools, gut models, and eventually human trials.

In conclusion, we propose that discovering and enabling specific substrate–microbe interactions is a valuable strategy to rationally design synbiotics that could establish a new category of hybrid nutra-/pharmaceuticals.

## Introduction

The gut microbiota emerges as a modulator of host health, as indicated by an increasing number of health disorders (including obesity, type 2 diabetes, allergic and autoimmune diseases, cancers, inflammatory bowel diseases, and brain disorders) that have been linked to a dysfunctional gut microbiota.^1^ This link appears to be bidirectional; therefore, microbiota-targeted strategies, including the application of prebiotics, probiotics, synbiotics, and fecal transplantations, have been conceived as novel therapeutic opportunities to prevent or treat these disorders.

**Figure 1.**
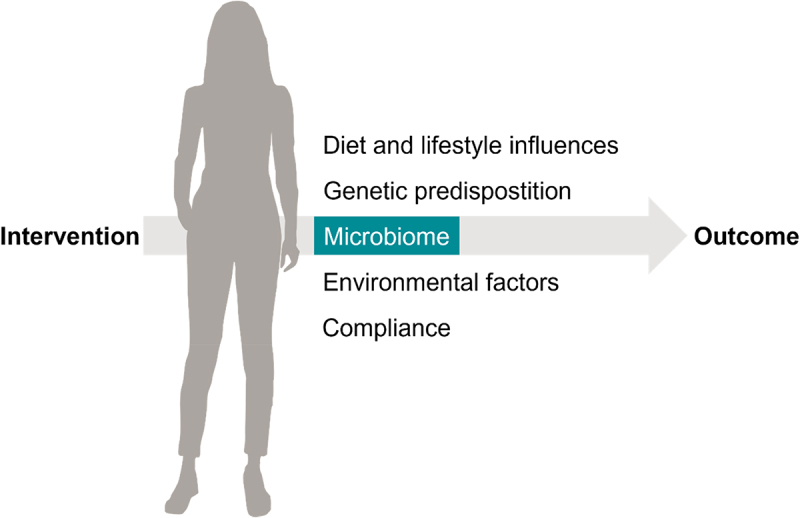
Important confounders in clinical trials.

The composition of the gut microbiota differs significantly between individuals and affects human and animal physiology via, for example, soluble factors derived from microbial metabolism, modulation of local and systemic immune cells, and modulation of the enteric nervous system and vagus nerve. On the other hand, gut microbiota composition and activity are affected by intrinsic (genome, gender, age, diseases) and a multitude of extrinsic factors, with diet probably being the most important determinant. The complexity of diet–microbiota interactions explains, to a large extent, the inter-individual variation observed in intervention studies with foods, food components, and drugs. As such, the gut microbiota is an important confounder in clinical trials (Figure 1) and, in some cases, argues for personalized or microbiota type-based therapies and nutritional advice.^2–4^ Well-known examples are the equol and urolithin phenotypes, which occur only in parts of the population and determine the fate of the precursors daidzein and ellagitannins, respectively, and thereby, the physiological response to plant foods containing these ingredients.^2,5^ The physiological relevance of food – microbiota interactions has long been known, and more recently, this knowledge is also being transferred to the use of orally administered drugs. Drug-microbiota connections are of high relevance, considering that the majority of drugs exhibit low solubility and permeability^6^ and are therefore likely to be exposed to the immense metabolic potential of small and large intestinal microbiota. A striking example of diet-drug-microbiota interactions has been provided by Tintelnot *et al*. showing that a microbial metabolite derived from the amino acid L-tryptophan has a specific supportive effect in the chemotherapy of pancreatic cancer.^7^

Besides diet and lifestyle, genetic predisposition, environmental factors and compliance to the protocol, the human microbiome is an important confounder in clinical trials. Interrelations of the gut microbiota with dietary and xenobiotic compounds can modulate their pharmacokinetics and dynamics as well as trigger indirect physiological effects.

Generally, interrelations between the gut microbiota and oral drugs can modulate their pharmacokinetics and dynamics, as well as trigger indirect physiological effects via changes in the microbiome. Such interrelations have often been overlooked, but in some cases, unveil relevant modes of action, such as microbiome modulation by metformin, statins, proton pump inhibitors, laxatives, ACE inhibitors,^8,9^ and the dietary compounds sodium chloride, selenium, and iron.^10–12^ On the other hand, (pro) drugs as well as phase I or phase II drug metabolites can be activated or inactivated by gut microbes, which is another contributing factor to response heterogeneity and the side effects of drugs. The biochemical reactions involved include oxidation, reduction, hydrolysis, acetylation, deacetylation, demethylation, deamination, and decarboxylation. Examples of cancer prodrug activation include CB1954, 5-fluorocytosine, banoxantrone, and fludarabine by probiotic *E. coli, Lactobacillus* and *Bifidobacterium* species.^13^ A case of unfavorable prodrug activation by gut microbes is the reactivation of hepatically conjugated (=inactivated) cancer drug irinotecan by β-glucuronidases, which can cause severe diarrhea, the major and dose-limiting side effect of this drug.^14^ Zimmermann *et al*. disentagled the contributions of host and gut microbial metabolism to the pharmacokinetics and toxicity of brivudine and clonazepam.^15^ This study, which is translatable to other drugs, clearly aids in understanding how the gut microbiome can determine the dosing and outcomes of drug treatments.

An example of a colon-targeted prodrug design is the inflammatory bowel diseases (IBD) drug sulfasalazine (SSZ), which is metabolized via azo cleavage by colonic bacteria to pharmacologically active 5-aminosalicylic acid (5-ASA).^16^ Since orally administered 5-ASA – but not SSZ – is absorbed in the small intestine, SSZ and other 5-ASA conjugates are suitable delivery forms for the treatment of colonic diseases. Similarly, the IBD prodrug azathioprine is converted by colonic bacteria (including *Enterococcus faecalis*, *Escherichia coli, Campylobacter concisus, Bacteroides fragilis, Bacteroides vulgatus*, and *Bacteroides thetaiotaomicron*) into 6-thioguanine nucleotides (6-TGN), which have local anti-inflammatory effects in the colon.^17^ Colon-targeting of thiopurines may, therefore, be applied specifically in IBD to minimize the side effects of systemic thiopurine treatment, as previously proposed.^18^ Colon-targeting *per se* has been achieved through the use of indigestible fibers (such as resistant-starch, pectin, chitosan, chondroitin, dextran, or guar gum) applied as coating materials that are degraded by gut bacteria.^19^

A classic example of drug inactivation by microbes is the reduction of the cardiovascular drug digoxin to dihydrodigoxin by *Eggerthella lenta*.^20^ Indirect modulation of drug efficacy can occur via microbial metabolites interfering with the host metabolic pathways involved in the activation and inactivation of drugs. Furthermore, the gut microbiota affects drug bioavailability via effects on drug transport, first-pass effect and enterohepatic cycling, bile acid modification, luminal pH, transit time, integrity, and thickness of the intestinal mucosa. The roles of probiotic strains as modulators of drugs are largely unexplored, with few studies performed in rodents, as reviewed by Zhang et al.^19^

Overall, substrate–microbiota interactions are still a relatively new field, but worthwhile to be explored further to predict and improve outcomes of treatments for various conditions not only restricted to the gastrointestinal tract. In the case of substrates being drugs, the interactions may translate to microbiome-based stratification of patients to existing drugs or prodrugs, the development of new microbiome-targeted or microbiome-preserving drugs, and the optimization of existing drugs by concomitant microbiome modulation (e.g., via probiotics or synbiotics). In the case of nutritional substrates, interactions with the microbiota can be used for personalized nutrition recommendations and the design of pre-, pro-, and synbiotic nutraceuticals.

## Microbial metabolism and role of metabolites

The human gut microbial metagenome comprises up to 22 million non-redundant genes,^21^ which is approximately one thousand times the number of genes in the human genome. This indicates the immense metabolic capacity of the gut microbiome, which is also reflected by more than 18,000 significant associations between microbial metabolic pathways and blood and fecal metabolites.^22^

Analyses of metabolomes derived from gut microbiota have been performed to gain insights into their genomic potential under different dietary interventions or other conditions. One must bear in mind that such realization is subject to multiple influencing factors, such as the accessibility of substrates provided through the diet, orally delivered drugs, excipients, environmental pollutants, and the use of microbiota-targeted drugs, as well as age, gender, and host genetics. In addition, especially in untargeted metabolome analysis, conditions of the analytical platform, such as sample processing, modification, specificity, and limits of detection methods, may curtail the assessment of certain metabolites.^23^

The contributions of (gut) microbiota to host metabolomes were assessed by comparing germ-free and flora-inoculated mice. In these studies, up to ~1,000 gut luminal metabolites were detected, depending on the analytical platform and inoculum used,^24–26^ the majority of which were affected by the presence of gut microbiota. Matsumoto *et al*. reported the occurrence of >100 colonic metabolites in germ-free versus SPF mice, as determined by capillary electrophoresis time-of-flight mass spectrometry, confirming the production of short-chain fatty acids (SCFA), gamma-amino butyric acid (GABA), and the polyamines putrescine and spermidine, as well as the clearance of uric acid.^25^ Intestinal colonization not only affects the luminal metabolome but also other host compartments, as shown by Moriya et al.^26^ in a multi-compartment metabolome analysis of germ-free and SPF mice. Between 20% and 30% of metabolites in the plasma, liver, and kidney were significantly changed, whereas changes were even higher in the urine and distal colon (>50% and 70%, respectively). Metabolites derived from different substrate classes (e.g., lipids, carbohydrates, amino acids, and xenobiotics) also display tissue-specific changes, which presumably reflect differences in their absorption, distribution, metabolism, and elimination. Attempts have also been made to unveil the microbiota-dependency of the human metabolome. An extensive phenotyping dataset (e.g., dietary records, human genotype, stool microbiome data) of 1,368 individuals from two Dutch cohorts was used to assign 1,183 plasma metabolites annotated by untargeted metabolic profiling to genetics, diet, and/or gut microbiome as determining factors.^27^ The latter was identified as dominant modifier of the plasma metabolome, explaining a larger proportion of interindividual variation (12.8%, without adjusting for covariates) than diet, intrinsic factors (age, sex, and body mass index), and smoking. A total of 610 and 85 plasma metabolites were classified as diet- and microbiome-dominant, respectively. Notably, the close interrelation between diet and microbiome was manifested here for 153 metabolites that were associated with both factors.

In the following section, we summarize the physiological functions and biosynthetic routes for a selection of auspicious microbial target metabolites. An overview is presented in Table 1.

**Table 1.** Production and functions of microbial metabolites derived from dietary or pharmaceutical compounds.

Substrate	Product(/s)	Effects	Microbes	Reference
Amino acids L-alanyl-L-glutamine Prebiotic carbohydrates	Butyric acid	Enterocyte differentiation and survival Lipid and glucose homeostasis	*Lachnospiraceae* *Ruminococcaceae* *Bacteroidetes*	31, 97
Anthocyanins	Protocatechuic acid Ferulic acid Phloroglucinaldehyde	Glucose homeostasis, redox regulation	*Lactobacillus* sp.	4, 5
Daidzein	Equol	Cardiovascular, Estrogenic (FMD, lipid metabolism)	*Eggerthellaceae* *Lactococcus garvieae* 20–92	62,63
Ellagitannins Ellagic acid	Urolithin A Urolithin B	Induction of autophagy Mitochondrial biogenesis Anti-inflammatory	*Gordonibacter* *Paraeggerthella* *Eggerthella*	2, 94
EPA DHA	Specialized pro-resolving mediators (SPM)	Resolution of inflammation	*Bacillus megaterium*	97
Flavan-3-ols	Phenyl-γ-valerolactones	Anti-inflammatory Anti-tumorigenic	*Lactiplantibacillus plantarum* IFPL935 *Eggerthella lenta* *Adlercreutzia equolifaciens* *Flavonifractor plautii*	73–75
Gliadin-epitopes Gluten	Short peptides Amino acids	Clearance of immunogenic or toxic epitopes	*Lactobacillus* *Bacillus* *Rothia*	144–146, 146, 147
L-arginine	Spermidine	Induction of autophagy	*Bacillus subtilis* *Weizmannia coagulans* YF1	64, 110
Lignans	Enterolactone Enterodiol	Modulation of estrogen signaling Modulation of lipid metabolism	*Bacteroides* *Clostridium* *Bifidobacterium* *Lactobacillus* *Eubacterium limosum* *Eggerthella lenta* *Blautia producta*	81
L-tryptophan	Indole-3-lactic acid Indole-3-acetic acid Indole-3-propionic acid L-kynurenine	Modulation of immunity Xenobiotic metabolism, Gut barrier integrity Arylhydrocarbon receptor signaling Pregnane-X receptor signaling	*Lactobacillus* sp.	144, 154
Xanthohumol,	8-prenylnaringenin	Estrogenic (bone formation, glucose homeostasis)	*Eubacterium limosum*	27, 82, 84

### Butyrate

Acetate, propionate, and butyrate are the major beneficial SCFA produced by gut bacteria in the large intestine. Butyrate is a common target molecule of interventions with prebiotics and/or probiotics, as it maintains gut health by strengthening intestinal barrier integrity (1) and triggering anti-inflammatory signaling.^28^ A fraction of the intestinally produced butyrate reaches the circulation and exerts systemic actions in the liver, adipose tissue, and pancreas, modulating glucose and lipid metabolism.^28,29^ Of note, systemic effects of butyrate are also triggered upon binding to G-protein coupled receptors located on intestinal epithelial and immune cells followed by signaling events with (also) extraintestinal output. Alterations in the butyrate-producing capacity of the gut microbiome have consequently been linked to chronic inflammatory and malignant diseases of the gut as well as to cardiometabolic diseases (reviewed in.^29,30^ The dominant butyrate-forming pathway for carbohydrates is the acetyl-CoA pathway involving butyryl coenzyme A (CoA):acetate CoA transferase as the main terminal enzyme, which is present in more than 20% of all gut bacteria.^31^ Most butyrate-producing taxa belong to *Lachnospiraceae* and *Ruminococcaceae*, including *Faecalibacterium prausnitzii*, *Oscillibacter*, and *Clostridium* group XIVa. Dietary strategies to elevate intestinal and/or systemic butyrate levels target these taxa through carbohydrate and peptide substrates and through direct application of probiotic strains of *Faecalibacterium prausnitzii*, *Clostridium butyricum*, *Clostridium beijerinckii*, and *Anaerobutyricum hallii*. The efficacy of these strategies remains to be determined in the case of probiotics and can be limited by the practice of exclusion diets and food intolerance issues.^32,33^

### L-tryptophan metabolites

**Figure 2.**
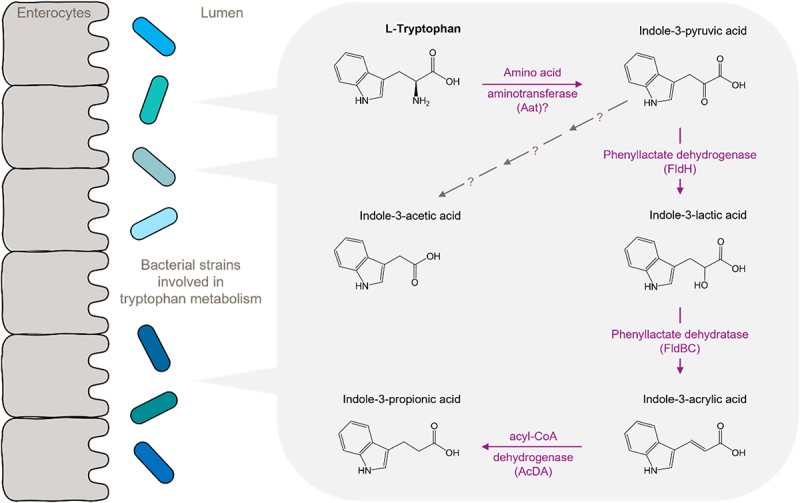
Microbial tryptophan metabolism by C. sporogenes.

L-Tryptophan (Trp) is an essential proteinogenic amino acid that is used for protein biosynthesis. In addition, human Trp metabolism yields nicotinic acid mononucleotide, nicotinamide dinucleotide, L-kynurenine (KYN), and serotonin. Non-proteinogenic Trp metabolism is also executed by gut microbes, which contribute to the fecal and circulating pool of Trp metabolites in mammals.^34^ Formation of these microbial metabolites appears to occur throughout the gastrointestinal tract, particularly in the stomach, small intestine, and large intestine.^34–36^ The existing literature remains rather vague regarding the contribution of the individual segments to the formation of the different metabolites. What is known is that Trp-metabolizing taxa are present in all of these segments, that their density increases toward the large intestine, whereas the concentration of available Trp is highest in the small intestine and reduces drastically beyond that.^37^ The same holds true for other amino acids and many other nutrients due to their rapid absorption in the small intestine. Overall, this implies that a triangle of luminal substrate availability, presence/engraftment of metabolizing microbes, and -if systemic availability is a prerequisite for an intended health effect – potential for absorption of microbially produced metabolites needs to be handled to set up efficient synbiotic intervention strategies. The most prevalent microbial metabolite of Trp is indole, which is released by the tryptophanase-expressing genera *Escherichia*, *Bacteroides*, and *Clostridium*.^38^ Notably, some Trp metabolites, including indole-3-lactic acid (ILA), indole-3-acetic acid (IAA), and indole-3-propionic acid (IPA), appear to be exclusively produced by gut microbes and not by host cells^34,36^ (Figure 2). The precursor of IAA and ILA is indole-3-pyruvic acid (IPyA), which forms from Trp, possibly by an aromatic amino acid aminotransferase. The route from Trp to IAA appears to be species-dependent and involves either an indolepyruvate decarboxylase and aldehyde dehydrogenase (IPyA pathway) or L-tryptophan decarboxylase-, diamine oxidase- and aldehyde dehydrogenase-catalyzed reactions.^35^ Phenyllactate dehydrogenase or indolelactate dehydrogenase (FldH) convert IPyA to ILA, followed by formation of indole-3-acrylic acid through phenyllactate dehydratase or indolelactoyl-CoA dehydratase (FldBC) and subsequent reduction to IPA by acyl-CoA dehydrogenase or indoleacrylate reductase (Figure 2). Other Trp metabolites, such as KYN, are derived from both human and microbial metabolism. In line with this, fecal levels of IAA and KYN are drastically reduced in germ-free mice compared with conventional mice.^36^ Therefore, the gut microbiota composition and dietary Trp availability are major determinants of the bioavailability of these compounds. Furthermore, xenobiotic Trp derivatives, such as the hallucinogenic candidate dru-gs N,N-dimethyltryptamine (DMT), 5-methoxy-N,N-dimethyltryptamine (5-MeO-DMT), and 5-hydroxy-N,Ndimethyltryptamine (bufotenine), have been produced from Trp by bioengineered *E. coli in vitro*. This study by Friedberg et al.^39^ exemplifies how the metabolic potential of bacteria can be tailored toward specific xenobiotic compounds for use as in-body pharmaceutical factories.

Tryptophan (Trp) is metabolized by an aminotransferase reaction, probably catalyzed by aromatic amino acid aminotransferase (Aat) as a first step. This enzyme activity has been demonstrated in C. sporogenes. Indole-3-pyruvic acid is converted to indole-3-lactic acid by phenyllactate dehydrogenase (FldH). Indole-3-lactic is then dehydrated to indole-3-acrylic acid by phenyllactate dehydratase (FldBC). Finally, indole-3-acrylic acid is reduced by acyl-CoA dehydrogenase (AcdA). For the pathway of indole-3-acetic acid production the enzymes involved remain unknown. Adapted from: Dodd et al., Nature. 2017;551(7682):648-652.

The health effects of microbial Trp metabolites have been inferred by mechanistic, animal, and human association studies. A recent study by Tintelnot *et al*. revealed a specific role for IAA in the response of pancreatic cancers to chemotherapy; IAA oxidation by myeloperoxidase produced 3-methylene-2-oxindole, leading to increased reactive oxygen species levels and diminished autophagy, which ultimately reduced the proliferation of pancreatic tumor cells.^7^ Additional Trp metabolites have been proposed to support chemotherapies of other cancers,^40^ e.g., a role for IPA in breast cancer, also via induction of oxidative stress in tumor cells.^41^ IPA, IAA, and KYN have been linked to psychological functions such as mood, appetite, and anxiety via effects on neuroinflammation, Trp uptake via the blood-brain-barrier, and Trp-to-serotonin metabolism.^42–44^ KYN in intestinal epithelial cells promotes expansion of mucosal RORγt (+)IL-22(+) ILC3 cells, which in turn stimulates proliferation of mucus-producing goblet cells, thereby supporting gut barrier integrity.^45^ ILA have been described to protect against inflammatory bowel diseases by modulating mucosal CD4+ T-cell differentiation.^46^ Some Trp metabolites are agonists of the aryl hydrocarbon receptor (AhR),^41,47–49^ a transcription factor that regulates the expression of genes involved in xenobiotic metabolism,^50^ immunity,^46^ and the expression of interleukin 22^48^ in various organs, including the liver, gut, lung, and brain. AhR thereby affects various health conditions, such as chronic inflammatory diseases of the gut (colitis), lungs (e.g., asthma bronchiale), and brain (e.g., major depressive disorder). Other Trp metabolites, such as IPA, reportedly engage the pregnane-X receptor, thereby regulating intestinal barrier function^51^ and inhibiting the proliferation of breast cancer cells.^41^ Similar to butyrate, Trp metabolites can exert extraintestinal effects upon reaching the circulation but also via signaling through effector cells located in the mucosa-like enterochromaffin cells, which in turn connect with vagal nerves.^43^

The gut microbial taxa involved in the formation of Trp metabolites include *Lactobacillus* sp., *Clostridium sporogenes*, *Clostridium botulinum*, *Bacteroides*, and *Escherichia*.^34,35,38,46,48^ *Clostridium sporogenes* produces IPA, ILA, and IAA (Figure 2), as well as PA, LA, and AA derivatives of the other aromatic amino acids phenylalanine and tyrosine, via the enzymes FldH, FldBC, and AcdA.^34,52^ A comprehensive investigation of the Trp-metabolizing potential of probiotic bacteria, as provided by a recent multi-omics analysis of lactic acid bacteria,^35^ will facilitate novel synbiotic compositions to achieve targeted Trp-dependent effects in the host. Bioengineered bacteria may be developed to produce additional xenobiotic Trp derivatives for pharmaceutical applications.

### Isoflavone daidzein metabolites

Soy isoflavones are naturally occurring phytoestrogens, which have structural resemblance with 17β-estradiol and are found mostly in the form of glycoconjugates in plants. After ingestion, they are hydrolyzed by gut bacteria into bioactive aglycones, including daidzein, genistein, and glycitein.^53^ The consumption of isoflavones has been suggested to reduce the risk of hormonal diseases, cardiovascular diseases, breast cancer, and postmenopausal osteoporosis.^54–56^ However, clinical studies have reported inconsistent results. Considering the metabolic fate of isoflavones, the gut microbiome is one of the major factors likely to account for this inconsistency.

**Figure 3.**
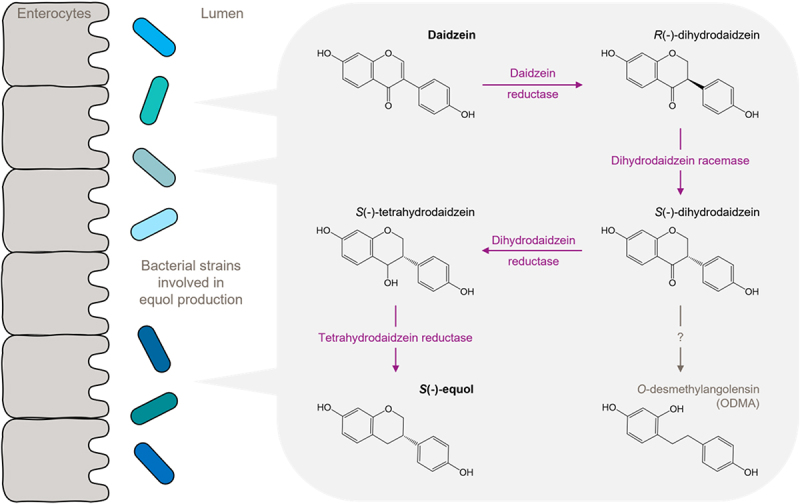
Microbial daidzein metabolism.

Equol and *O*-desmethylangolensin (ODMA) are metabolites of the isoflavone daidzein, with the four enzymes daidzein reductase (DZNR), dihydrodaidzein racemase (DDRC), and dihydrodaidzein reductase (DHDR), and tetrahydrodaidzein reductase (THDR) being directly involved in equol production (Figure 3), while the route from S(-)-dihydrodaidzein to ODMA remains to be described. The production of equol or ODMA indicates the presence of specific gut bacterial taxa that influence the ability to metabolize daidzein, leading to the stratification of individuals into equol-, ODMA-, and non-producer phenotypes.^5^ In Caucasian populations, a large proportion of adults convert daidzein to ODMA, and only 25–30% of adults are identified as equol-producers. The percentage of equol-

Specific gut bacteria can metabolize daidzein. The first step in this biochemical pathway is a reduction to *R*(-)-dihydrodaidzein, followed by a racemization to *S*(-)-dihydrodaidzein and further reductions to *S*(-)-tetrahydrodaidzein and subsequently to *S*(-)-equol. The enzymes responsible for the transformation of *S*(-)-dihydrodaidzein to *O*-desmethylangolensin (ODMA) are not yet fully understood. ODMA and equol pathways are provided by different bacterial taxa. Adapted from: Mayo et al., Nutrients. 2019;11(9):2231.

producers is much higher in Asian populations consuming soy-rich diets, reaching 50%−60%.^53,57^ The stability of the daidzein metabolite phenotype and whether it is affected by diet remain to be shown.

Hitherto, most of the evidence suggests that equol producers are a more favorable phenotype for health. Among the 1345 postmenopausal women, the equol-producing group had a significantly lower percentage of participants with metabolic syndrome than the equol non-producing group (6.6% vs. 10.9%).^58^ Two population studies supported that, compared with equol non-producers, the equol-producing phenotype was associated with a lower risk of aortic calcification and coronary artery calcification.^59^ Metabotypes are also likely to be used to predict obesity prevalence, with an association found between obesity and non-producers of ODMA in both peri- and postmenopausal women.^60^ Furthermore, an RCT found that equol producers gained more cardiometabolic benefits from a 4-week isoflavone-rich diet than equol non-producers, including significantly reduced serum total cholesterol (TC), low-density lipoprotein cholesterol (LDL-C), hsCRP, and flow-mediated dilation (FMD) compared with baseline values.^61^

The gut microorganisms involved in the biotransformation of daidzein to equol are mostly from the Eggerthellaceae family, including *Adlercreutzia equolifaciens*, *A. mucosicola*, *Slackia_A isoflavoniconvertens*, *Slackia_A equolifaciens*, *Enteroscipio* sp000270285, and *Lactococcus garvieae* 20–92.^62,63^ Other equol-producing bacteria have been identified in the *Bifidobacteriaceae* and *Coriobacteriaceae* family (including *Asaccharobacter* and *Eggerthella*) and *Clostridium* genus.^64,65^ However, investigations into the gut microbiome involved in the conversion of daidzein to ODMA are still limited.

The administration of *S*-equol supplements has been investigated in several observational and RCTs. In an RCT with Japanese overweight or obese adults, 12 weeks of daily ingestion of 10 mg *S*-equol significantly decreased HbA1c and serum LDL-C.^66^ However, acute intake of *S*-equol supplements did not confer any vascular benefits to male equol non-producers.^67^ Other studies have shown that equol supplements improve menopausal symptoms and bone health.

### Flavan-3-ols and related metabolites

Flavan-3-ols, which are among the most consumed polyphenols, are abundant in tea, red wine, berries, and cocoa. Studies have linked flavan-3-ol consumption to improved vascular function, serum cholesterol concentration, and blood glucose metabolism. In double-blinded randomized controlled trials, flavan-3-ol intake reduced CVD risk and improved endothelial function in both healthy adults and patients with type 2 diabetes.^68–70^ In healthy adults, the improvement in FMD can be predicted by certain plasma polyphenol metabolites; for example, 3’-hydroxycinnamic acid and glucuronide metabolites were predictors of 1-month FMD effects.^69^

2,3-dihydroxybenzoic acid (DHBA), a flavan-3-ol (and more generally, many other polyphenol subclasses) metabolite with ascertained functions, has shown beneficial effects on blood glucose control and lipid metabolism.^71^ Phenyl-γ-valerolactones (PVLs) and 3-(hydroxyphenyl)propanoic acids (HPPs) are also flavan-3-ol metabolites, and their production shows large inter-individual variability.^72^ Some people produce high amounts of PVLs and limited amounts of HPPs, whereas others produce opposite results. However, information regarding the gut microbiome species involved in flavan-3-ol metabolism is limited. To date, *Lactiplantibacillus plantarum* IFPL935, *Eggerthella lenta*, *Adlercreutzia equolifaciens*, and *Flavonifractor plautii* have been shown to be involved in the biotransformation of flavan-3-ols into 5-(3′,4′-dihydroxyphenyl)-γ-valerolactone and 5-(3′-hydroxyphenyl)-γ-valerolactone.^73–75^ The species responsible for the production of phenylvaleric acids and HPPs remains unknown. Further studies are needed to investigate this further to target the intestinal production of these metabolites.

### Lignan metabolites

Lignans are phytoestrogens, as they have (weak) estrogen-like activity, and they also have anti-inflammatory and antioxidant properties.^76^ Enterolactone (EL) and enterodiol (ED), also known as enterolignans, are major gut microbial metabolites of dietary lignans. Enterolignans, particularly EL, have been shown to modulate estrogen signaling and lipid and bile acid metabolism.

The health benefits of enterolignans have been confirmed in epidemiological and interventional studies. Data from 4685 US adults in the National Health and Nutrition Examination Survey showed a significant interactive association between diet quality and lignan gut metabolites for various cardiometabolic risk factors, including triglycerides, LDL cholesterol, HDL cholesterol, SBP, DBP, insulin, and oral glucose tolerance.^77^ A crossover RCT found that a high lignan-to-EL conversion phenotype was linked to anti-inflammatory status, indicated by the inhibition of NF-κB and NOS2 and upregulation of PPARγ.^78^ In cohort studies, both EL and ED have been found to be inversely associated with obesity or weight gain.^79^

**Figure 4.**
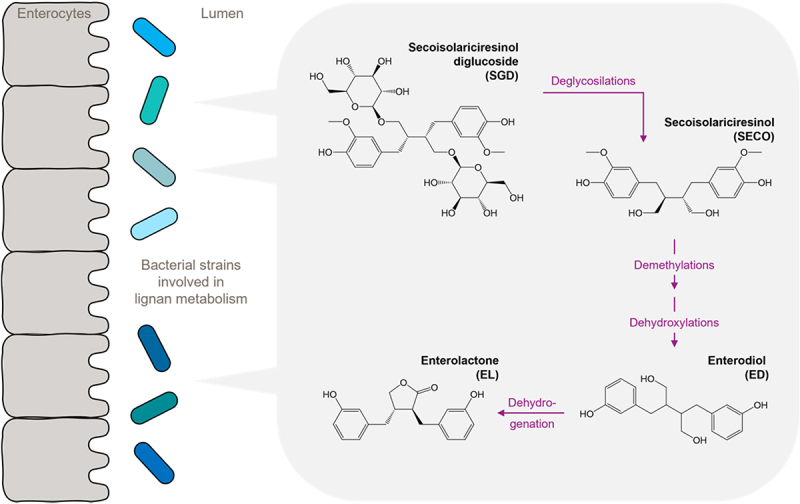
Microbial lignan metabolism.

The metabolism of dietary lignans into enterolignans requires four steps (Figure 4). Upon ingestion, dietary lignans are reduced to secoisolariciresinol diglucoside (SDG).^80^ Gut bacteria including *Bacteroides distasonis*, *Bacteroides fragilis*, *Bacteroides ovatus* and *Clostridium cocleatum*, as well as the newly isolated strain *Clostridium* sp. SDG-Mt85-3 Db are responsible for the biotransformation of SDG to secoisolariciresinol (SECO) through deglycosylation reactions, then SECO undergoes demethylation (through *Butyribacterium methylotrophicum*, *Eubacterium callanderi*, *Eubacterium limosum* and *Peptostreptococcus productus*) and dihydroxylation (*Clostridium scindens* and *Eggerthella lenta*) to generate ED.^80^ A crucial step in generating EL is dehydrogenation of ED, which occurs fast and is thought to be mediated by ED-Mt61/PYG-s6.^81^ This process is likely to explain the high inter-individual variability in ED and EL production observed in human studies.

Upon ingestion, dietary lignans are reduced to secoisolariciresinol diglucoside (SDG). The four steps of the microbial metabolism of dietary lignans to enterolignans are the following: Gut bacteria transform SDG to secoisolariciresinol (SECO) via deglycosylation reactions. Then SECO undergoes demethylations and dihydroxylations to generate Enterodiol (ED). Finally, ED is dehydrogenated to Enterolactone (EL).

### Hop prenylflavonoids metabolites

Xanthohumol, isoxanthohumol (IX), and 8-prenylnaringenin (8-PN) are prenylflavonoids found in hops and beers, of which 8-PN is a potent phytoestrogen and a possible treatment for postmenopausal symptoms. In vitro and in vivo studies have revealed that 8-PN acts as an estrogen receptor agonist and regulates bone formation and glucose homeostasis.^82^ IX has also been found to ameliorate insulin resistance through modification of the gut microbial ecology and suppression of the inflammation response.^83^ The gut bacterium Eubacterium limosum can convert IX into 8-PN,^84^ but also other bacteria with methyltransferase activity such as Eubacterium ramulus are able to catalyze this reaction and transform 8-P into the chalcone derivatives O-desmethylxanthohumol (DMX) and O-desmethyl-α,β-dihydroxanthohumol (DDXN).^85^ The same authors performed comprehensive microbiota experiments in vitro and in vivo and showed that the introduction of this species was sufficient to transmit an 8-PN producer phenotype into rats. The potential of this species as a probiotic to promote 8-PN production and further health-related benefits in humans still need to be confirmed.

### Urolithins from ellagitannins

**Figure 5.**
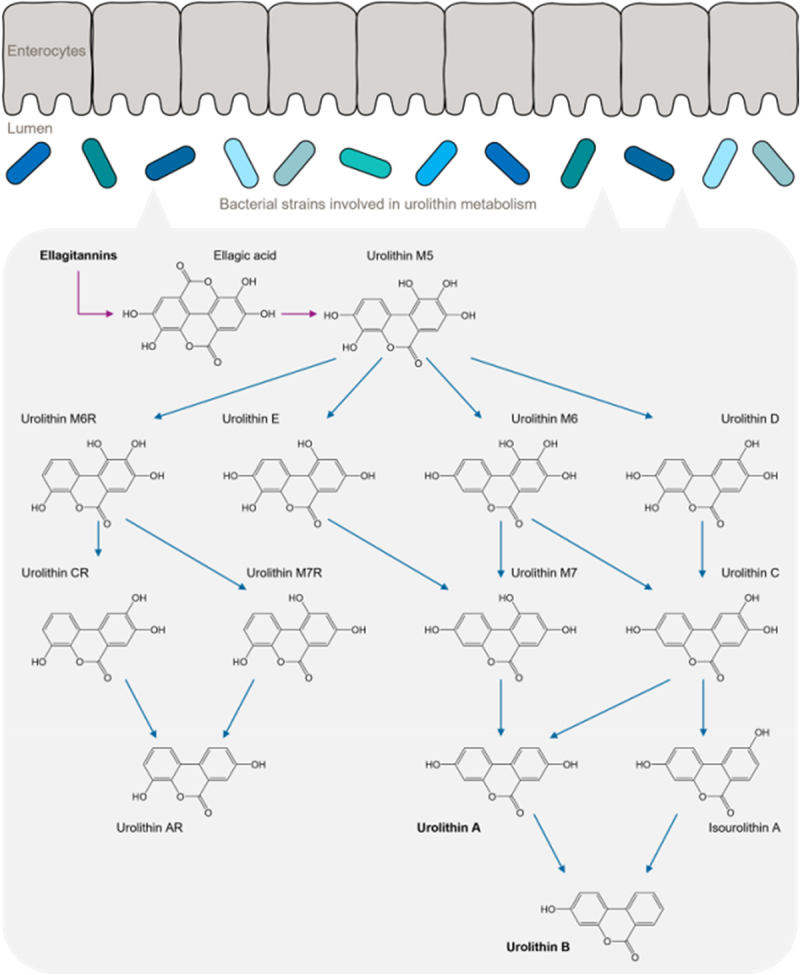
Microbial urolithin metabolism.

Urolithins are gut microbial metabolites derived from ellagitannins and ellagic acid, which are polyphenolic compounds widely abundant in our diet, particularly in nuts and fruits, such as strawberries, raspberries, and pomegranates. Recent studies conducted with ellagitannin-rich foods have indicated that the gut microbiota may be a key factor in explaining the beneficial health effects of such foods. It has been hypothesized that the differences in the metabolism of ellagitannins within individuals can be clustered into three different phenotypes for urolithin production in the gut: urolithin metabotype A (UMA), which produces urolithin A; UMB, which produces urolithin B and isourolithin A, in addition to urolithin A; and UM0, which is unable to produce any urolithin metabolite^86–88^ and that these differences may have important consequences for their health benefits.^2,86,87^ The biochemistry of urolithin metabolism starting from Urolithin M5 consists of a series of dehydroxylations, as depicted in Figure 5.

Ellagic acid, which is first released from ellagitannins, is converted to urolithin M5. Urolithin M5 was then stepwise dehydroxylated from five OH groups down to one OH-group. Dehydroxylation is indicated by blue arrows. The first dihydroxylation step resulted in compounds with four OH groups around the urolithin basic structure: urolithin M6R, urolithin E, urolithin M6, and urolithin D. The second dihydroxylation step resulted in the 3-OH compounds urolithin CR, urolithin M7R, urolithin M7, and urolithin C. The third dihydroxylation step yields urolithin AR, isourolithin A, and urolithin A with two OH-groups groups. The final dihydroxylation leads to urolithin B with a 1 OH-group. Adapted from García-Villalba et al. Molecular Nutrition & Food Research. 66(21), e2101019. *Gordonibacter urolithinfaciens*, *Gordonibacter pamelaeae, Clostridium coccoides* members, *Ellagibacter isourolithinifaciens* and *Bifidobacterium pseudocatenulatum* are among the newly identified bacteria responsible for the conversion from ellagitannins to urolithins.

Ellagic acid, which is first released from ellagitannins, is converted to urolithin M5. Urolithin M5 was then stepwise dehydroxylated from five OH groups down to one OH-group. Dehydroxylation is indicated by blue arrows. The first dihydroxylation step resulted in compounds with four OH groups around the urolithin basic structure: urolithin M6R, urolithin E, urolithin M6, and urolithin D. The second dihydroxylation step resulted in the 3-OH compounds urolithin CR, urolithin M7R, urolithin M7, and urolithin C. The third dihydroxylation step yields urolithin AR, isourolithin A, and urolithin A with two OH-groups groups. The final dihydroxylation leads to urolithin B with a 1 OH-group. Adapted from García-Villalba et al. Molecular Nutrition & Food Research. 66(21), e2101019.

The distribution of UM has been reported in a Spanish cohort, with approximately 50–80% UMA, 10–40% UMB, and 10% UM0, distribution which was found to be highly dependent on age, with UMA being more frequent at early ages than UMB, which was found to be more prevalent in older individuals.^89^ For UM, the overall evidence available from a few small studies suggests that UMA may be a more favorable metabotype than UMB and UM0 in terms of weight management. Women with the UMA phenotype lost weight and restored their microbiome one-year postpartum, whereas women with UMB did not.^90^ In addition, UMB and UM0 were found to be risk factors for obesity in children.^91^ Information on which bacteria can metabolize (poly)phenols and the differences in the gut microbiome between metabotypes is scarce.

The gut microbial species responsible for producing different urolithins have been identified. *Gordonibacter pamelaeae* and *G. urolithinfaciens* from the *Eggerthellaceae* family can convert ellagic acids into urolithin M5, M6, and C.^92,93^ The genus *Gordonibacter* was positively correlated with Urolithin A production and HDL-cholesterol, whereas it was inversely correlated with urolithin B and isorolithin A production.^93^ On the other hand, *Ellagibacter isourolithinifaciens* from *Eggerthellaceae* family can produce urolithin M5, M6, C, and A from ellagic acids.^93^ Differences in the gut microbial ecology between urolithin metabotypes have also been detected. While the genera *Gordonibacter*, *Paraeggerthella*, and *Eggerthella* were correlated with the UMA phenotype, the genera *Ellagibacter*, *Olsenella*, *Senegalimassilia*, *Slackia*, and *Adlercreutzia* were correlated with UMB.^94^ This explains the inter-individual variations in ellagitannin and ellagic acid metabolism and the subsequent health effects. Further studies are warranted to investigate the health benefits of these bacteria and ways to promote their growth.

To date, two RCT have investigated the safety, tolerability, and bioactivity of urolithin A supplementation. In the first study conducted in healthy older adults, Urolithin A was found to be bioavailable in plasma, and 4 weeks of daily treatment with 500 mg and 1,000 mg of urolithin A modulated plasma acylcarnitines and skeletal muscle mitochondrial gene expression.^95^ The second study showed that supplementation with Urolithin A significantly increased plasma levels, and Urolithin A producers were distinguished by a significantly higher gut microbiome diversity and ratio of *Firmicutes* to *Bacteroides*. Clearly, the F/B ratio is a superficial parameter of microbiome composition, coming from a time when the possibilities of bioinformatic analysis were far from the current state of the art, and the significance of this parameter is low. Futures urolithin A studies will benefit from including deeper functional and compositional microbiome analyses.

### Specialized pro-resolving mediators

Specialized pro-resolving mediators (SPM) comprise specific oxygenation products of omega-3 fatty acids (eicosapentaenoic acid (EPA), docosahexaenoic acid (DHA)) and omega-6 fatty acids (arachidonic acid (AA)), referred to as maresins (MaR), E- and D-series resolvins (RvE and RvD), protectins, lipoxins, and precursors, such as 18-hydroxy-eicosapentaenoic acid (18-HEPE), 17-hydroxy- docosahexaenoic acid (17-HDHA), and 17,18-epoxyeicosatetraenoic acid (17,18-EEQ). SPM are endogenously formed by lipoxygenases (LOX), cyclooxygenase-2 (COX-2), and cytochrome P450 monooxygenases (CYP450) and act as potent agonists of active inflammation resolution.^96^ Consequently, the application of SPM has been effective in a multitude of infectious and inflammatory disease models, summarized in,^97^ whereas translation into the clinic has thus far been limited, for example, to the use of the resolvin E1 (RvE1) analog RX-10045 in allergic conjunctivitis and ocular inflammation (NCT01639846 and NCT02329743). The common approach toward increasing SPM levels in humans is via ingestion of EPA/DHA (the precursors of SPM) through diet or dietary fish oil supplements. Importantly, this strategy is hampered by the fact that the SPM-producing machinery is dysfunctional under certain conditions, as indicated by the reduced (local or circulating) SPM levels in diabetic wounds, metabolic syndrome, asthma, ulcerative colitis, Crohn’s disease, and periodontitis (referenced in,^97^ as well as the reduced expression or activity of SPM-producing enzymes, such as severe asthma,^98^ ulcerative colitis,^99^ and periodontitis.^100^ These studies explain the overall lack of benefit of omega-3 interventions, especially for patients with IBD, asthma, and metabolic syndrome.^101–103^ One possible approach is to target the omega-3/-6)-to-SPM conversion rate in vivo. This can be achieved via modulation of the enzymes involved, as exemplified by allosteric activation of 15-LOX-1 by 3-O-acetyl-11-keto-β-boswellic acid (AKBA),^104^ and in humans upon lipopolysaccharide (LPS) endotoxin challenge that elevated SPM levels in omega-3-supplemented healthy subjects.^105^ Alternatively, gut microbes may compensate for insufficient eukaryotic potential to produce SPM. The possibility of such a route was supported by the identification of LOX and CYP450 genes in bacteria.^106,107^ The occurrence of oxygen-consuming enzymes in gut-residing microorganisms is limited; cyclooxygenases and lipoxygenases appear to be absent in nonpathogenic gastrointestinal bacteria and archaea. CYP450 monooxygenases have been detected in Bacillus.^107^ CYP102A1, also known as CYP450BM–3, is a bifunctional enzyme found in Priestia megaterium that catalyzes the NADPH-dependent hydroxylation of polyunsaturated fatty acids via consecutive oxygenase and reductase activities. Purified CYP450BM–3 was shown to generate 18-HEPE, the precursor of RvE1, from EPA,^108^ and More recently, we provided evidence that Priestia megaterium produces a broad and strain-specific spectrum of SPM, presumably determined by highly variant CYP450BM–3 gene sequences.^97^ Gut microbial SPM production is a very recent topic, and therefore, unlike the previous examples of poly(phenol) metabolism, the occurrence of omega-3 metabotypes is thus far unknown. It may be worthwhile to reanalyze existing microbiome data for presence of Priestia megaterium − general proof of existence in human feces has been provided^109^, CYP102A1 or other omega-3 oxygenation genes or transcripts and correlate this with omega-3 intakes and clinical features linked to inflammation (resolution). The location of microbial SPM production is an important consideration; duodenal production will compete with absorption of precursors (EPA/DHA) but may use the same transport mechanisms as for fatty acids. On the other hand, colonic production may be particularly beneficial to achieve intestinal effects as in the context of ulcerative colitis.

### Polyamines (spermidine, spermine, putrescine)

**Figure 6.**
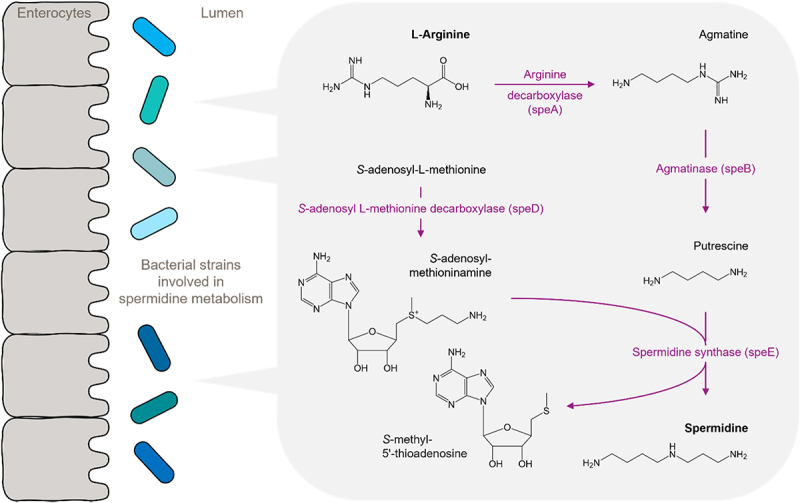
Spermidine metabolism of Bacillus subtilis.

Spermidine is a natural polyamine that occurs in high amounts in semen, as well as in other eukaryotic cell types and body fluids, as well as in wheat germ and some other foodstuffs.^110^ It has been characterized as a „caloric restriction mimetic,” which mediates the beneficial effects of intermittent or chronic fasting.^111^ The main mode of action of spermidine is the induction of cytoprotective autophagy, which promotes the removal of dysfunctional proteins, aggregates, and organelles, thereby contributing to the maintenance of homeostasis at the cellular, tissue, and organ levels. This vital activity explains the beneficial effects of spermidine supplementation on longevity and health span across species including yeast, *C. elegans*, flies, and mice. In humans, dietary spermidine intake is correlated with decreased mortality.^112^ Intriguingly, spermidine levels decline during aging, which suggests correcting this decline either by direct supplementation^113^ or stimulation of endogenous production through precursor substrates and/or probiotics. The latter strategy seems plausible, considering that the (cecal) microbiota may contribute more to circulating spermidine levels than diet.^114^ Microbial spermidine-producing taxa include *Bacteroides*, *Fusobacterium, Bacillus subtilis OKB105*, and *Weizmannia coagulans* YF1.^115^ Spermidine biosynthesis starts with L-arginineand involves the genes speA (arginine decarboxylase), speB (agmatinase), speE (spermidine synthase), and speD (S-adenosyl L-methionine decarboxylase) as depicted in Figure 6. Intracellular spermidine can be acetylated by speG, bltD, or secreted via transporters mdtI and mdtJ, and in this way becomes available to the host.^110,116^ Similarly to SPM, typing gut microbiota for their capacity to produce spermidine has thus far not been done on a larger scale. Assessing this capacity in larger cohorts may help understand if and how microbial spermidine is correlated with or determines e.g., aging, and similar traits linked to spermidine.

L-arginine decarboxylation by speA leads to agmatine. SpeB cleaves agmatine to putrescine, which is then converted to spermidine by SpeE. This reaction involves the cofactor S-adenosyl-methioninamine, which is formed via the decarboxylation of S-adenosyl-methionine by speD.

## Development and clinical testing of novel substrate–microbe interactions

### A differentiated definition of synbiotics

Numerous trials with probiotics have been used in combination with non-digestible fermentable carbohydrates, such as fructo-oligosaccharides (FOS), galacto-oligosaccharides (GOS), arabinoxylan-oligosaccharides (AXOS), xylo-oligosaccharides (XOS), beta-glucans, and complex human milk oligosaccharides (HMO),^117^ which are collectively referred to as *prebiotics*. The most recent definition of and motivation for use of a prebiotic is that it is “a substrate that is selectively utilized by host microorganisms conferring a health benefit” (International Scientific Association for Probiotics and Prebiotics (ISAPP).^118^ The ISAPP further defines *synbiotics* as “a mixture comprising live microorganisms and substrate(s) selectively utilized by host microorganisms that confers a health benefit on the host”,^119^ whereby *host microorganisms* shall include resident as well as externally administered microorganisms. In both the definition of *prebiotic* and *synbiotic* the prebiotic is understood to serve as a supporter of health-beneficial microorganisms – probiotics or resident microorganisms – by selectively promoting their growth, activity, or engraftment, whereby the *selectivity of utilization* with regard to specific taxa is an object of debate.^120,121^

**Figure 7.**
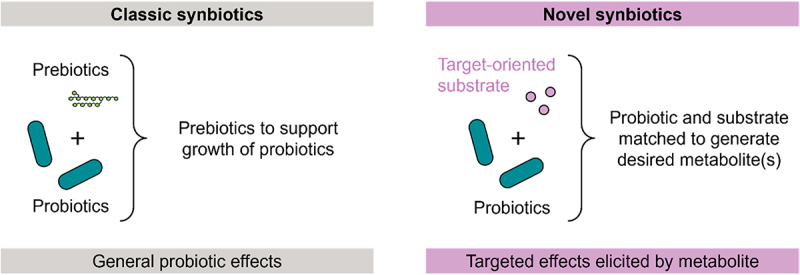
A differentiated definition of synbiotics, comparing classic versus metabolite-mediated modes of action.

**Figure 8.**
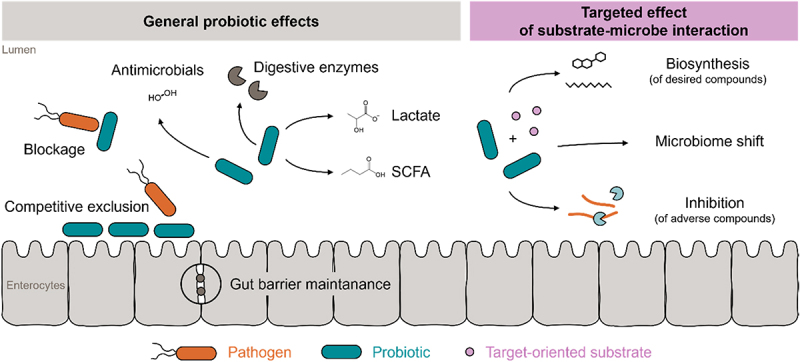
Comparison of general probiotic effects with targeted substrate–microbe interaction.

In the context of this review, we changed the perspective of conventional probiotic/prebiotic interactions, considering that among the many modes of action of probiotics, or more general microbes, the microbial metabolism is a powerful but so far undervalued one. We understand that microbial metabolism is a very useful biological tool for the targeted enzymatic conversion of substrates, leading to the gain of beneficial function or loss of adverse function molecules. More precisely, we here introduce a metabolism-focused definition of the term *synbiotic*, or *metabiotic-based synbiotic*, as combinations of microbial strains (single strains or consortia) with any kind of defined substrate(s) (isolated or contained in a food matrix or contained in the diet) that synergize toward targeted microbial metabolism of such substrate(s) (Figure 7). This metabolism is achieved either by the applied probiotic/s directly or indirectly via a modifying effect on the gut microbiota of the host. The product of metabolism may then exert a specific physiological effect in the host (the compound serves as a prodrug) or has lost the toxicity of the educt (the compound being detoxified) (Figure 8). This metabolism-focused understanding of synbiotics integrates into a broader understanding of it, defining synbiotics as: “combinations of microbial strains (single strains or consortia) with any kind of defined substrate(s) (isolated or contained in a food matrix or contained in the diet) that synergize toward achieving a health benefit on the host”, and in that sense includes all modes of action, not only the metabiotic one, whereby probiotics modulate health. According to this new definition, the substrate(s) may be used by the strains to support their growth, activity, engraftment, and/or toward their targeted metabolism.

Left: Classic definition of synbiotics as a mixture comprising live microorganisms (probiotics) and prebiotics selectively utilized by host microorganisms that confers a health benefit on the host, with prebiotics serving as substrates for growth of the probiotics or beneficial resident microbes. Right: novel definition of synbiotics as target-oriented combination of metabiotic probiotics with specific substrates to generate desired metabolites.

Left: The general probiotic modes of action include the digestive support in breaking down fiber, the production of lactate and short-chain fatty acids, which are important energy sources for enterocytes strengthening the gut barrier and the immune defense, activity against pathogenic bacteria by competitive exclusion, blockage, or the production of antimicrobials or inhibitors of quorum sensing, and immune modulation via microbial antigens. Right: Targeted mode of action: Substrate-microbe-interaction, resulting in *in vivo* biosynthesis of desired compounds, microbiome modulation, or inhibition of adverse compounds.

Using the metabolism-focused definition, a few metabiotic-based synbiotic combinations have been tested *in vivo*. Prebiotic carbohydrates and (poly)phenols are the most prevalent classes of ingredients that have been assessed as compounds, followed by proteins and amino acids. The genera *Lactobacillus* and *Bifidobacterium*, as well *Bacillus, Streptococcus, Escherichia coli, Propionibacterium*, and *Enterococcus* are commonly used as probiotics. Many of these genera have long been used in food production (e.g., yogurt, cheese, sourdough-derived baked products, Sauerkraut, or Natto), food fortification, and in the dietary supplement industry. It is therefore no surprise that the same taxa have been chosen for synbiotic attempts; however, this restriction limits the possibilities of achieving synbiotic effects, as shown in more detail below, and the subsequent health benefits.

### The case for synbiotic substrate–microbe interaction: in vivo production versus direct application of purified microbial metabolites or postbiotics

Some products of microbial metabolism are available in the form of food or food preparations; for example, spermidine is available as a dietary supplement in the form of dried wheat germ extract.^113^ Furthermore, metabolites can be applied directly as purified substances, as is the case for butyric acid, which is available as an ingredient in dietary supplements or orally or rectally applied drugs in the form of sodium or calcium butyrate.^122,123^ However, for most cases, the direct application of a purified microbial metabolite is either not possible or faces some major technical and consumer compliance challenges. Food ingredients as well as active pharmaceutical ingredients need to comply with national legislation, which in most cases, unless included in a positive list, means that the safety and/or efficacy of the (novel) ingredient has to be documented in an extensive approval process, as, for example, laid down in Regulation (EU) 2283/2015, which has not been done for the majority of cases discussed here. Substance stability during storage in formulated products and during gastrointestinal transit can also be an issue. SPM, for example, is susceptible to further oxidation and has a short half-life *in vivo*,^124,125^ they are usually administered topically, parenterally, or in the form of stabilized derivatives in clinical and animal trials^126,127^ (NCT00799552). Microbial enzymes, as contained in digestive aid supplements, are prone to proteolysis in the stomach and small intestine, and therefore have a short duration of action, limiting their efficacy and user-friendliness.^128,129^ Many microbial metabolites have an unpleasant odor or taste (e.g., short-chain fatty acids or polyamines), thus requiring odor/taste masking efforts or limiting the doses applied. An additional drawback of direct applications is the unfavorable pharmacokinetic profile upon sudden release of substances from the carrier, which is disadvantageous compared to sustained substance release or formation thereof.

It is possible to apply microbial metabolites in crude mixtures, such as in the form of bacterial lysates or extracts, as postbiotics. However, postbiotics face the challenge of composition variability, affecting efficacy, specificity, and safety; a lack of defined mechanisms, delivery challenges, and poorly developed regulatory frameworks.

### Examples of synbiotic combinations for in vivo production of beneficial target metabolites

An overview of the substrate-microbe couples tested *in vivo* is presented in Table 2.

**Table 2.** Substrate-microbe couples tested in vivo.

Substrate	Microbe	Effects	Reference
Flavanones	*Bifidobacterium longum* R0175	Increased excretion of flavanone metabolites after chronic intake (Shown in humans)	136
EPA DHA ARA	*Priestia megaterium* DSM 32,963	Increased plasma levels of 5-HEPE, 15-HEPE, 18-HEPE, 4-HDHA and 7-HDHA (Shown in humans)	11
L-arginine	*Bifidobacterium animals subsp. lactis*	Increased serum levels of putrescine and spermidine (Shown in humans)	143
Gluten	*Lactiplantibacillus plantarum* DSM33363 *Lactiplantibacillus plantarum* DSM33364 *Lacticaseibacillus paracasei* DSM33373 *Limosilactobacillus reuteri* DSM33374 *Priestia megaterium* DSM33300 *Bacillus pumilus* DSM33297 *Bacillus pumilus* DSM33355	Gluten degradation (Shown in humans)	147
Cadmium	*Lactiplantibacillus plantarum* CCFM8610	Blood cadmium removal (Shown in mice)	152
Copper Nickel	*Pediococcus acidilactici* GR-1	Blood copper removal Blood nickel removal (Shown in humans)	153

### Equol

Equol production is an attractive strategy for synbiotic applications. Attempts using the generic probiotic strains *Lactobacillus acidophilus* DDS1 and *Bifidobacterium longum* failed to induce equol levels in soy-supplemented women.^130,131^ Before the identification of specific daidzein-metabolizing strains, an equol-producing microbial consortium was isolated from a human fecal sample and was found to transfer its phenotype to microbiota from non-equol-producing individuals *in vitro*.^132^ However, neither this consortium nor full fecal transplants have been tested for their metabolic competence in humans. The fact that such consortia are difficult to specify and manufacture on a larger scale is clearly a drawback to their possible application as a dietary supplement or drug, even more so in a preventative setting. The recent discovery of an equol gene cluster, comprising a set of eight core genes, and its distribution within human gut metagenomes^62^ allows for a more straightforward search and use of equol-producing strains in synbiotic applications. The cluster is highly conserved in the *Eggerthellaceae* family but also appears in unrelated taxa, for example, *Slackia_A equolifaciens*, *Enteroscipio* sp000270285, and *Lactococcus garvieae* 20–92.^62^

Finding this cluster, or at least the four genes directly involved in equol production (DZNR, DDRC, DHDR, and THDR) within typical probiotic taxa, e.g., *Lactococcus lactis*, *Lactobacillus* sp., *Bifidobacterium* sp. would significantly accelerate its possible application in humans. In fact, *Bifidobacterium breve* 15700 and *Bifidobacterium longum* BB536^133^ are reportedly capable of metabolizing daidzein to varying degrees to equol, although the existence of the equol gene cluster in these strains and their performance within a complex gut microbiota remains to be confirmed. A *Lactobacillus sp*. Niu-O16 indirectly supported *Eggerthella sp*. Julong 732-dependent equol production *in vitro*, possibly via a prebiotic or cross-feeding effect of *Lactobacillus* strain.^134^

Recently, Kydd et al. developed a successful equol bioengineering approach with a proof-of-concept *in vivo*.^135^ They produced *E. coli* Nissle strains with ectopic expression of the genes required for daidzein-to-equol conversion (DZNR, DDRC, DHDR, and THDR) cloned from the *Lactococcus garvieae* 20–92 strain mentioned above.^62^ These four genes were split into two complementary *E. coli* Nissle P1 and P2 strains to obtain a higher product yield. Administration of the strains to soy-fed mice led to roughly 3 times higher serum equol levels than soy feeding alone. This study provides a good example of the prospects of synbiotic approaches beyond common probiotic features. Nevertheless, synbiotic daidzein-equol conversion remains to be successfully implemented in humans, and similar to the limitations of diverse microbial consortia, genetically modified microbes face regulatory hurdles and therefore have a long path to the food or drug market. A quicker translation may be seen upon the discovery and validation of equol gene cluster-positive taxa familiar to the market and with a history of safe use.

### Flavanones and probiotics

Flavonoids are a promising class of compounds for synbiotic strategies given the array of microbial metabolites with physiological relevance derived from them (e.g., ferulic acid).

Pereira-Caro *et al*. assessed the metabolic fate of orange juice flavanones upon supplementation with *Bifidobacterium longum* R0175 in healthy humans fed an otherwise polyphenol-low diet.^136^ Although lacking a placebo control, this study is interesting as it shows that long-term (4 weeks) intake of the probiotic increases (orange juice) polyphenol bioavailability, determined per polyphenol metabolite content in 24 h urine samples, compared to a single concomitant intake. The impact of the strains on bioavailability appears to be indirect, possibly via a proposed microbiome-modulatory effect, and is not mediated by a specific catabolic function. This unknown mode of action makes it difficult to ascertain strain-specific functionality or benefits to the host. In a similar approach, modulation of polyphenol bioavailability and microbial metabolism was observed for a combination of cranberry extract (mainly comprising procyanidins, flavonols, and anthocyanins) with a *Bacillus subtilis* CU1 strain. Chronic co-intake of the extract and strain significantly increased plasma levels of total phenolic metabolites compared to the extract-only group, with elevation of the metabolites *p*-/m-coumaric acid and p-hydroxybenzoic acid. Similar to the study involving *Bifidobacterium longum* R0175, the metabolome pattern cannot be directly linked to an enzymatic function of the strain because the catabolism of flavonoids involves enzymes provided by multiple taxa of the resident gut microbiota, and treatment-induced microbiome modulation may have led to the change in flavonoid metabolism. Most food polyphenols are glycosides, and their (microbial) deglycosylation improves absorption and initiates further microbial metabolism. An array of microbial deglycosylases are required to release these aglycones, which offers a starting point for the selection of polyphenols by probiotics expressing suitable enzymes. For example, kaempferol was released from kaempferol-3-sophoroside by *Lacticaseibacillus paracasei* 221, and synbiotic feeding in mice increased kaempferol plasma levels compared to kaempferol-3-sophoroside feeding alone.^137^ Generally, it is important to determine the robustness of the effects of synbiotic combinations on different baseline microbiota; if the composition is a strong determinant, large inter-individual (or diet-, disease-, age-specific) variation is unlikely to be overcome by the treatment.

### Butyrate

We developed a synbiotic composition of *Bacillus subtilis* DSM 32,315 and L-alanyl-L-glutamine (Ala-Gln), which increases gut microbial butyrate levels *in vitro* and in humans when administered orally via a capsule with colon-targeted release.^138^ Colon targeting was applied to avoid duodenal uptake of the dipeptide and thereby increase its availability to butyrate-producing bacteria, which are mainly localized in the colon. The composition derived from a screening of different *Bacillus* species to act as probiotic microbiome modulators toward the expansion of known butyrate-producing taxa, as has been shown previously for the genus *Bacillus*.^139,140^

**Figure 9.**
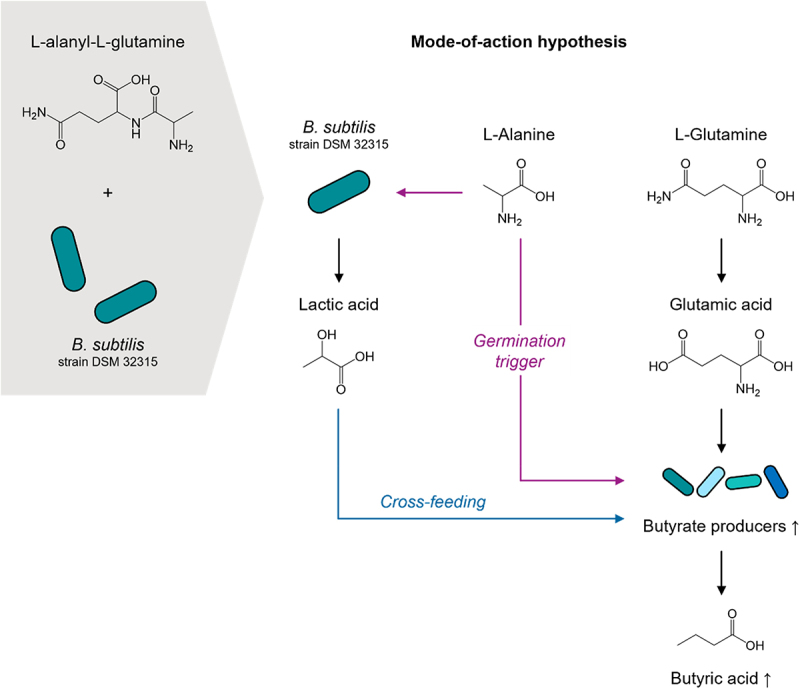
Mode-of-action hypothesis for a synbiotic combination of Bacillus subtilis DSM 32,315 and Ala-Gln.

Ala-Gln was chosen from a set of non-fibrous substrates (amino acids and peptides) that feed into catabolic pathways toward butyric acid.^31^ The L-glutamine residue is catabolized to butyric acid via the acetyl-CoA butyrate pathway, whereas it is stabilized in the dipeptide Ala-Gln by L-alanine, which may also serve as a spore germination trigger (Figure 9). Both components led to an expansion of Clostridium group XIV, including *Faecalibacterium prausnitzii* in gut simulation models, which was also recapitulated in a pilot trial upon supplementation with a synbiotic combination of *Bacillus subtilis* DSM 32,315 and Ala-Gln together with additional ingredients, including vitamin B_12_, an essential cofactor of the acetyl-CoA butyrate pathway.^138^ The microbiome-modulating effect of *Bacillus subtilis* DSM 32,315 was discussed in that study to involve cross-feeding of lactic acid produced by *Bacillus subtilis* and used by butyrate-producing taxa. Synbiotic approaches toward the elevation of intestinal and circulating butyrate levels, though in their infancy, are worthwhile to develop further, also with regard to the thus far disappointing clinical outcomes of novel butyrate-producing probiotics^32,141^ and the limited applicability and/or effectiveness of fiber supplementation for certain subgroups.^142^

The combination of *Bacillus subtilis* strain DSM 32,315 and L-alanyl-L-glutamine (Ala-Gln) can upon intake increase gut microbial butyrate producers and fecal butyrate levels. *B. subtilis* promotes butyrate-producing taxa via cross-feeding of lactate, while L-alanine may serve as a germination trigger. The L-glutamine residue is metabolized by butyrate producing taxa toward butyric acid.

### Specialized pro-resolving mediators

**Figure 10.**
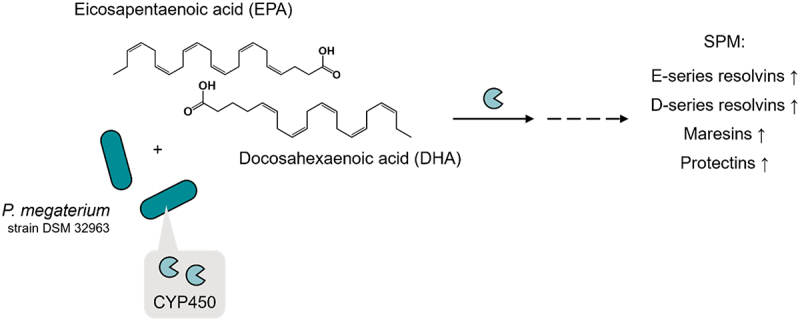
*Priestia megaterium* and omega-3 fatty acid synbiotic combination.

As outlined in Section 2, existing omega-3 interventions often fail to provide clinical benefits in chronic inflammatory diseases, which may be due to the insufficient SPM production capacity of the host organism. A synbiotic-based strategy could overcome these drawbacks by acting as a self-sufficient SPM production tool in the gastrointestinal lumen. Depending on the localization of SPM production, this strategy can be particularly useful in the treatment of gastrointestinal diseases (irritable bowel syndrome and inflammatory bowel diseases) and/or applied to increase circulating SPM levels. The description of an 18-HEPE-producing CYP isoform found in Priestia megaterium^108^ prompted us to search for putative SPM-producing probiotic bacteria suitable for a synbiotic formulation.^97^ We found that Priestia megaterium per se can convert *n*-3 PUFA in the form of free fatty acids (FFA) to a broad spectrum of SPM in a strain-specific manner, and this variation is presumably controlled by polymorphisms of the CYP450BM–3 gene^97^ (Figure 10). A synbiotic capsule product combining Priestia megaterium DSM 32,963 and *n*-3 PUFA FFA lysine salt was prepared to serve as a self-sufficient SPM-producing formulation. In a human pilot trial, four-week consumption of the synbiotic led to significantly increased plasma levels of several SPM precursors (*n*-3 PUFA monooxygenation products 5-HEPE, 15-HEPE, 18-HEPE, 4-HDHA, and 7-HDHA) in healthy humans. It will now be worthwhile to test this synbiotic for efficacy in chronic inflammatory diseases, particularly inflammatory bowel diseases, in comparison with standard (non-microbial) *n*-3 PUFA interventions. Furthermore, Priestia megaterium strain-specific SPM profiles offer the potential to target select conditions that would benefit most from certain types or profiles of SPM.

*Priestia megaterium* strains (e.g., *Priestia megaterium* DSM 32,963) have enzymatic equipment to convert EPA and DHA to a broad spectrum of SPM and their precursors.

### Spermidine

A straightforward strategy for microbial spermidine production was followed by Caffarati et al.^116^ through metabolic engineering of the *Escherichia coli* Nissle 1917 strain (EcN), similar to the equol example mentioned above. Ectopic expression of speE and speD was sufficient to increase spermidine production and secretion by transformed EcN. The introduction of speA and speB genes was not required, as putrescine is already a metabolite of wild-type EcN. Still, putrescine formation was a limiting factor for spermidine production, as shown by its further elevation in putrescine-supplemented transformed EcN. Interestingly, a large proportion of spermidine was maintained intracellularly as mono- and diacetylated spermidine. This was counteracted by overexpression of the spermidine exporter MdtI/MdtJ, which elevated spermidine levels in supernatants of speE/speD-transformed cells by roughly threefold. This study clearly shows that the availability of a metabolite to the host depends not only on substrate availability and microbial metabolic potential but also on the control of transport mechanisms and alternative biosynthetic pathways.

Supplementation of healthy humans with yogurt comprising *Bifidobacterium animals subsp. lactis* and L-arginine significantly increased serum putrescine and spermidine levels.^143^ Compared to placebo, yogurt intake also led to improved (i.e., increased) endo-peripheral arterial tone, a marker of reactive hyperemia, and a risk factor for arteriosclerosis. As single treatment arms (probiotic only, L-arginine only) were not included, a synergism between both components could not be clearly inferred. Nevertheless, this study links spermidine induction with a relevant cardiovascular outcome.

### Examples based on targetable substances with adverse functions

#### Gluten/Gliadin epitopes

Gluten is the main protein network in cereals such as wheat, rye, and barley. Gluten includes monomeric α-gliadins, β-gliadins, γ-gliadins, and Ω-gliadins, which carry peptide sequences such as the 33-mer LQLQPFPQPQLPYPQPQLPYPQPQ-LPYPQPQPF, positions 57–89 of α2-gliadin, with immunogenic and/or toxic potential. The incomplete digestion of gliadins can release these peptides, leading to adverse reactions in susceptible individuals, particularly with celiac disease (CD). CD pathology is triggered by gluten epitopes translocating the intestinal epithelium and causing a T-cell-mediated immune response in the small intestinal lamina propria. Accordingly, gliadins are the target structures for pharmacological and nutritional interventions in gluten-related disorders.^144^ Microbial proteases are particularly useful tools for gliadin (epitope) clearance given the high proline content of these peptides and the lack of prolyl-specific proteases in the human genome.^145^

**Figure 11.**
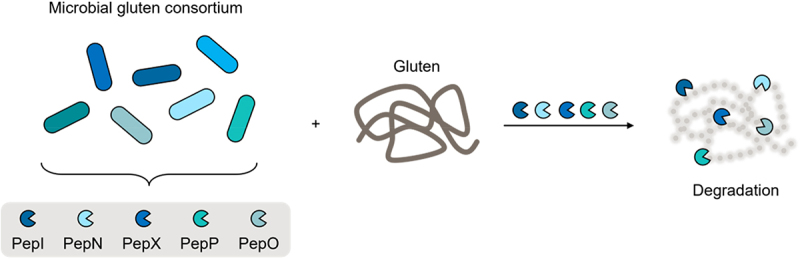
Microbial consortium for gluten degradation.

The commercial use of peptide hydrolases to detoxify gluten during food processing and directly in the human gut^129^ has been described. However, related products containing these enzymes have minimal evidence of efficacy; moreover, incomplete digestion triggered by such enzymes may pose a potential hazard in celiac disease. The peptidases general aminopeptidase type N (PepN), proline iminopeptidase (PepI), X-prolyl dipeptidyl aminopeptidase (PepX), endopeptidase (PepO), and prolyl endopeptidase (PepP) comprise a minimal enzymatic portfolio required for hydrolyzing the 33-mer and other gluten-derived immunogenic peptides.^146^ Recently, a gluten-degrading microbial consortium derived from a screening of *Lactobacillus* sp., *Bacillus* sp., *Weissella* sp., and *Pediococcus pentosaceus* for gastrointestinal survival and complementary activities of these five peptidases has been identified.^147^ The consortium hydrolyzed gluten and its derived key epitopes under simulated gastrointestinal digestion of bread into non-immunogenic digests (Figure 11). Importantly, digestion of bread under the same conditions with either *Aspergillus niger* prolyl endoprotease (AN-PEP) or mixed *Bacillus* protease-containing preparations caused only partial hydrolysis of gluten (epitopes) and elicited a marked immune response in *ex vivo* small-intestinal cultures from patients with CD (unpublished). A microbial consortium (comprising *Lactiplantibacillus plantarum* DSM 33,363, *Lactiplantibacillus plantarum* DSM 33,364, *Lacticaseibacillus paracasei* DSM 33,373, *Limosilactobacillus reuteri* DSM 33,374, *Priestia megaterium* DSM 33,300, *Bacillus pumilus* DSM 33,297 and *Bacillus pumilus* DSM 33,355) also degraded gluten in humans, as shown by De Angelis *et al*. in a randomized placebo-controlled trial of healthy participants receiving defined amounts of gluten (50–10 g gluten per day) on the background of a gluten-free diet (unpublished). While the consortium was viable and active under simulated stomach and small intestinal conditions, it will be important to confirm that this also holds true *in vivo*, and thus enable gluten epitope clearance before they can possibly translocate from the lumen into the small intestinal mucosa. It would also be worthwhile to test the efficacy of this consortium as adjuvant therapy for symptomatic patients with CD.

A composition of 7 bacterial strains (*Lactiplantibacillus plantarum* DSM 33,363, *Lactiplantibacillus plantarum* DSM 33,364, *Lacticaseibacillus paracasei* DSM 33,373, *Limosilactobacillus reuteri* DSM 33,374, *Priestia megaterium* DSM 33,300, *Bacillus pumilus* DSM 33,297 and *Bacillus pumilus* DSM 33,355) provides 5 peptidases (PepI, PepN, PepX, PepP and PepO) able to completely degrade gluten.

### Toxic heavy metals: lead, cadmium, mercury

According to the WHO, lead exposure is a significant public health concern (https://www.who.int/publications/i/item/9789240037656). Some lactic acid bacterial strains are capable of sequestering lead and other heavy metals in the gastrointestinal tract through absorption, precipitation, complexation, or chelation, thereby reducing systemic heavy metal exposure and toxicity.^148^ These functions have been attributed to gram-positive bacteria and are mediated in part by their cell wall components, including teichoic acid and peptidoglycans. The identification of heavy metal-binding microbes mostly follows *try-and-see* approaches; for example, the composition and contribution of the extracellular matrix are difficult to predict using bioinformatics and cell culture tools. A *Leuconostoc mesenteroides* L-96 strain showed lead resistance and binding capacity, which led to reduced lead poisoning of the testes and kidneys and increased fecal elimination in rodents^149^; similar observations were reported for *Lactiplantibacillus plantarum* strains CCFM8661, CCFM8610, and LP33.^150,151^ The Cd-lowering effect of *Lactiplantibacillus plantarum* CCFM8610 in mice was explained by an intriguing and strain-specific modulation of the enterohepatic circulation of Cd, which enhanced hepatic bile acid production and biliary Cd release, whereas ileal reuptake was inhibited through suppression of the enterocyte FXR-FGF15 axis.^152^ This modulation of enterohepatic bile acid-dependent cycling contributes significantly to the Cd excretion capacity of *Lactiplantibacillus plantarum* CCFM8610, as well as to *Lactiplantibacillus plantarum* strains with related Cd-binding capacity but lacking the BA-dependent mode of action.^152^ Several other rodent studies have shown clearance of cadmium and mercury by other lactic acid bacteria strains, mainly *Lactiplantibacillus plantarum*, through metal-probiotic interaction and excretion via feces, summarized in.^148^

Very few human trials have been conducted to test the mitigation of heavy metals by probiotics in populations affected by environmental heavy-metal pollution. *Lactiplantibacillus plantarum* CCFM8610 significantly reduced blood Cd levels in a heavy metal-exposed Hunan Province, China. *Pediococcus acidilactici* GR-1 provided in yogurt reduced blood copper and nickel levels in occupational workers from the metal industry,^153^ whereas *Lactobacillus rhamnosus* GR-1-containing yogurt slightly reduced blood mercury and arsenic levels in pregnant women, but not in schoolchildren from Tanzania. In conclusion, microbial heavy metal clearance involves several modes of action, whereby mediators and metal-specificity of these actions remain to be fully understood and may provide opportunities to counteract Cd and Pb intoxication, which needs to be replicated in larger studies.

### Uric acid

Some studies have indicated that probiotic and synbiotic approaches may help to treat hyperuricemia. Modes of action include the microbial conversion of uric acid (UA) into allantoin, a more soluble and easily excretable compound, elevation of fecal uric acid excretion via induction of intestinal epithelial excretory UA transporters, and inhibition of xanthine oxidase in the liver and serum of rats.^154^ Engineered EcN strains expressing purine- or UA-degrading enzymes ameliorate hyperuricemia in *Drosophila melanogaster* and rodents.^155^ In a study of hyperuricemic gout patients, a *Lactobacillus salivarius* CECT 30,632 strain derived from screening for purine-degrading efficacy was tested against the standard gout drug allopurinol and resulted in a significant reduction in the number of gout episodes.^156^

## Conclusions and outlook

Microbiome-targeted strategies have been investigated for their potential to prevent and treat prevalent diseases including type 2 diabetes, allergic and autoimmune diseases, cancers, inflammatory bowel diseases, and brain disorders. In the past and present, the success of probiotic interventions has been limited by the performance of the probiotic in terms of viability and functionality *in vivo* – as well as by the significant variability in gut microbiome composition, diet, and lifestyle among individuals. In addition, probiotics have typically been applied in the context of general gut health, and the huge diversity of protocols and tools for assessing gut health and disease makes it difficult to compare the efficacies of different interventions.

The term *synbiotic* in its common understanding refers to combinations of probiotics and prebiotics, whereby the two entities ideally provide some kind of synergy through which the potential for therapeutic intervention is higher than that for the single entities. In most cases, probiotics that had been previously tested or established for certain physiological effects were combined with prebiotic fibers, conveying either similar physiological effects or intended to act as substrates for the growth and engraftment of beneficial bacteria. Although there is a growing body of evidence to support the use of some synbiotic combinations, there are also several challenges that limit the clinical and nutritional impact of synbiotics and argue for a refined approach in their development. The limitations are the following:

- Lack of rationale: The efficacy and safety of synbiotics can vary greatly depending on the type, dosage, and formulation of probiotics and prebiotics used. There have been try-and-see approaches testing synbiotics for various clinical conditions, often resulting in unsatisfactory or inconclusive outcomes. In many cases, the modes of action of probiotics, as well as their activity under gastrointestinal conditions and possible interactions with their prebiotic counterparts, are poorly described.
- Heterogeneity of patient populations: The response to synbiotics can vary depending on various patient factors, such as gut microbiome, diet, medication, underlying health conditions, age, ethnicity, and lifestyle. Identifying and addressing the most relevant confounders are challenging.
- Limited clinical evidence: While there is growing evidence to support the use of synbiotics in various clinical conditions, the quality of evidence is often limited by small sample sizes, lack of standardization, and methodological limitations. Large-scale, well-designed clinical trials are needed to better understand the potential benefits and risks of synbiotics in different patient populations and clinical contexts.
- Regulatory challenges: The regulatory landscape for synbiotics is complex and varies by country, even more so for probiotics. Currently, there is no consensus on the classification and regulation of synbiotics, which can create uncertainty for manufacturers and limit their availability in certain markets. Regulatory hurdles hinder the use of unconventional but potent microbial taxa and limit the scientific and industrial community to a narrow group of established probiotics, such as those belonging to the genera *Lactobacillus*, *Bifidobacterium*, and *Bacillus*. Further, the European Food Safety Authority (EFSA) evaluates health claim applications made on foods, and EFSA explicitly asks for strong evidence for a plausible mechanism of action to substantiate such claims. General probiotic modes of action are considered too vague in that context, which is one reason why EFSA has so far rejected all health claim applications made on specific bacterial strains.

Therefore, we discuss refined approaches toward the development of microbiome-targeted intervention strategies, which are based on a differentiated understanding of the term synbiotic, that is, considering the intestinal microbiome as a potent and modifiable pharmaceutical factory that utilizes the metabolic capacity of microbes (“metabiotics”) as catalysts for converting nutritional and xenobiotic substrates into health-beneficial or detoxified target compounds. Several novel synbiotics have been developed and, in some cases, have also been tested in humans. However, there are few examples to judge the success of this strategy. In many cases, such synbiotics include polyphenols, as this group is particularly diverse and abundant in the human diet. However, an increasing body of evidence has uncovered interactions between other dietary (e.g., fatty acids, amino acids, or peptides) and xenobiotic compounds and the gut microbiome, which can also be used to develop nutraceuticals with lower response heterogeneity and personalized nutritional guidance.

Regardless of the compound, some technical considerations refer to all cases of novel synbiotics: the effectiveness of synbiotic compositions is dependent on several technical aspects, as the probiotic part must execute the intended metabolic activity under the conditions occurring *in vivo* at the target site in the GIT. This requires the manufacturability of the probiotic strain(s) as well as storage stability within the formulation and delivery format. Both aspects can be challenging, particularly for strict anaerobic conditions. In addition, substrates need to maintain their stability in a co-formulation, or may otherwise be ingested separately. The physical proximity of substrates and microbes providing the required enzymatic activities is mandatory and can be facilitated by targeted or delayed release formulation technologies or microbe-specific uptake and activation of substrates/derivatives thereof. For all active ingredients of a synbiotic formulation, targeted delivery techniques may be required to achieve metabolism at specific sections of the GIT, for example, by applying pH-sensitive, time-dependent, or prebiotic delivery systems for enteric or colon-targeting, or toothpastes, chewing gums, etc., to target the oral cavity. Enteric and colon-targeted delivery can be used to protect sensitive components against low pH and bile acids and to prevent substrate absorption in the small intestine. The luminal availability of a target metabolite is affected by microbial import and export mechanisms, subsequent metabolism, or post-translational modifications (e.g., acetylation of spermidine). The aspect of subsequent metabolism within a complex microbiota has been examined using microbial genome-scale metabolic modeling (GSMM), such as AGREDA, AGORA, or AGORA2,^157,158^ which represents the metabolic capabilities of intestinal microbial communities. GSMM can be used to simulate the metabolic fluxes of nutrients, drugs, xenobiotics, and microbial interactions under different environmental conditions, such as oxygen levels, pH, and host factors. The behavior and robustness of the intended substrate-microbe interactions can be analyzed by such models to help design synbiotic compositions for either select metabotypes or functionalities that are independent of the resident microbial community. GSMM predictions can then be validated using *in vitro* technologies (e.g., SHIME®, TIM-1, and TIM-2) that account for the spatial and temporal variations in the gut microbiota along the intestinal tract. Another approach is to use machine learning algorithms that can identify patterns and associations between gut microbiota composition and function and host outcomes such as weight loss, disease risk, or response to interventions. Furthermore, the systemic availability of metabolites depends on their transport mechanism via the mucosal barrier.

In conclusion, we discussed a new scientific concept based on a broader understanding of the term synbiotic, which focuses on the metabolism of substrates by gut microbes. This concept integrates into the classic understanding of synbiotics, required that the term prebiotic is understood in a broader sense and encompasses any substance that may serve not only for the growth, activity, and engraftment of probiotics, but also as a substrate for the formation of active ingredients or otherwise a toxin or allergen that can be detoxified by the probiotic or metabiotic. In a broader ecological sense, the microbiome-health connection implies that a diverse, functionally active microbiota in conjunction with a balanced diet offers the best chances of positively influencing health. Specific aspects of health and physiology may consequently be addressed by fine-tuning of this connection through select synbiotics with targeted modes of action. While the components of such synbiotic compositions are in most cases considered as food or dietary ingredients, their interactions and outcomes most likely also have a pharmacological implication, and this again shows how blurry the line is between food and medicine.

It is worthwhile to apply this concept to precision nutrition and precision medicine approaches, as it may ultimately lead to more consistent and predictable clinical outcomes. Furthermore, synbiotic compositions derived from this concept may advance the field of microbiome-targeted interventions by providing a more targeted mode of action that is less prone to inter-individual variability. Clearly, further novel synbiotics need to be developed and tested in comparison with conventional probiotics and prebiotics to judge the value of the concept described here.

## Data Availability

Data sharing not applicable – no new data generated.
